# Enzymatic Synthesis of Extremely Pure Triacylglycerols Enriched in Conjugated Linoleic Acids

**DOI:** 10.3390/molecules18089704

**Published:** 2013-08-13

**Authors:** Yu Cao, Weifei Wang, Yang Xu, Bo Yang, Yonghua Wang

**Affiliations:** 1 School of Bioscience and Bioengineering, South China University of Technology, Guangzhou 510006, China; 2 College of Light Industry and Food Sciences, South China University of Technology, Guangzhou 510640, China

**Keywords:** conjugated linoleic acid, mono- and di-acylglycerol lipase, hydrolysis, triacylglycerols

## Abstract

This work was objectively targeted to synthesize extremely pure triacylglycerols (TAG) enriched in conjugated linoleic acids (CLAs) for medical and dietetic purposes. Extremely pure CLA-enriched TAG was successfully synthesized by using the multi-step process: TAG was primarily synthesized by lipase-catalyzed esterification of CLA and glycerol and then the lower glycerides [monoacylglycerol (MAG) and diacylglycerol (DAG)] in the esterification mixtures was hydrolyzed to free fatty acids (FFAs) by a mono- and di-acylglycerol lipase (lipase SMG1), finally, the FFAs were further separated from TAG by low temperature (150 °C) molecular distillation. The operation parameters for the lipase SMG1-catalyzed hydrolysis were optimized using response surface methodology based on the central composite rotatable design (CCRD). The operation parameters included water content, pH and reaction temperature and all of these three parameters showed significant effects on the hydrolysis of lower glycerides. The optimal conditions were obtained with a water content of 66.4% (w/w, with respect to oil mass), pH at 5.7 and 1 h of reaction time at 19.6 °C. Under these conditions, the content of lower glycerides in the reaction mixture decreased from 45.2% to 0.3% and the purity of CLA-enriched TAG reached 99.7%. Further purification of TAG was accomplished by molecular distillation and the final CLA-enriched TAG product yielded 99.8% of TAG. These extremely pure CLA-enriched TAG would be used for *in vivo* studies in animals and humans in order to get specific information concerning CLA metabolism.

## 1. Introduction

Conjugated linoleic acid (CLA), referring to a heterogeneous group of geometrical and positional conjugated isomers of linoleic acid (LA, 18:2), has received increasing attention because of its demonstrated biological properties, including cancer inhibition [1,2,3,4], blood cholesterol lowering [5], diabetes controlling and weight loss effects [6,7]. Most of these studies, however, were carried out with CLA as free fatty acid (FFA) [4,5,6,7] and only a few studies have used CLA in the form of triacylglycerols (TAG) [8,9]. This is mainly because the CLA as FFA was the only material readily available, while CLA in TAG form is hard to be obtained because of its low purity, with lower glyceride [monoacylglycerol (MAG) and diacylglycerol (DAG)] contaminants. Since the nutritional profile and rheological behaviors of lipids depends on both their fatty acid composition and acylglycerol composition [10], the presence of these lower glycerides will impede the further understanding of nutraceutical effects of CLA in TAG form. Thus, in order to get specific information concerning CLA metabolism, it is necessary to synthesize highly pure TAGs enriched in CLA.

CLA-enriched TAG can be synthesized chemically or enzymatically through esterification or transesterification processes [11,12,13]. Compared with chemical methods, lipase-catalysed esterification provides an alternative process due to its mild performance conditions, which prevents CLA from being destroyed. Nevertheless, the purity of TAG products produced through esterification is always less than desirable because of the presence of lower glycerides. High temperature molecular distillation (220–250 °C) is a universally used technique to purify TAG products containing MAG and DAG. However, it is not suitable for the purification of CLA-enriched TAG, because it would lead to temperature-induced isomeration and thus the formation of unwanted CLA isomers. Thus, to attempt the purification of CLA-enriched TAG would be a challenging task.

In this paper, we aim to produce extremely pure CLA-enriched TAGs by a multi-step process. Firstly, the CLA-enriched TAG was synthesized by lipase-catalyzed esterification of CLA and glycerol, and then the lower glycerides (e.g., MAG and DAG) in the esterification mixtures were further hydrolyzed to FFA by lipase SMG1 (a novel mono- and di-acylglycerol lipase) and the hydrolysis process was optimized by the response surface methodology (RSM). Finally, the CLA-enriched TAG was easily separated by molecular distillation at a lower temperature (150 °C). This method enables the synthesis of extremely pure CLA-enriched TAG for medical and dietetic research purposes.

## 2. Results and Discussion

### 2.1. Lipase-Catalyzed Synthesis of CLA-Enriched TAG

**Figure 1:**
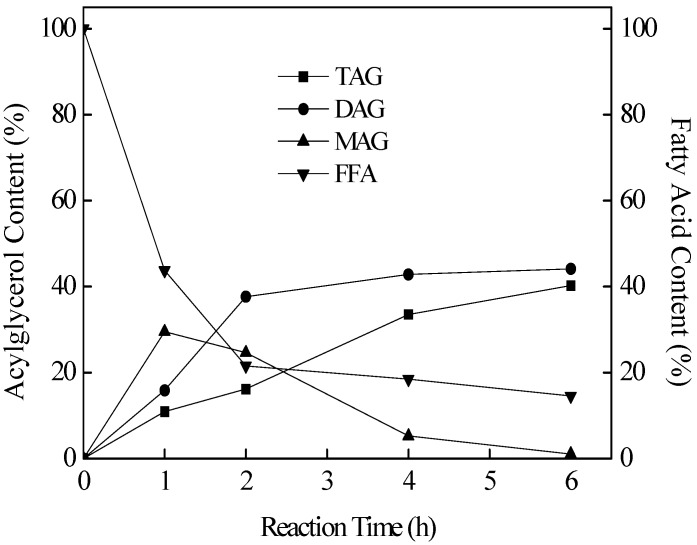
Novo 435 catalyzed esterification of CLA and glycerol. Reaction conditions were as follows: CLA/glycerol molar ratio, 3:1; enzyme loading, 1% (w/w, with respect to total reactants); temperature, 60 °C; pressure, 0.1 kPa; reaction time, 6 h.

CLA-enriched TAG was produced by Novozym 435-catalyzed esterification of glycerol and CLA under vacuum. The reaction was performed under the condition of a CLA/glycerol molar ratio of 3:1, an enzyme load of 1% (w/w, with respect to total reactants), at 60 °C and 0.1 kPa for 6 h. The results are shown in Figure 1. The content of TAG rose gradually to 40.24% after 6 h of reaction, while the DAG content increased dramatically at the first 2 h but then remained stable at around 40%. The content of MAG, however, increased sharply at first 1 h and then decreased slowly to around 1% at the end. After a total reaction time of 6 h, esterification product contained 40.24% TAG, 14.56% FFA, 44.14%DAG and 1.06% MAG were achieved. The FA composition of the TAG fraction in the esterification product was analyzed and the CLA content in the TAG fraction reached to 80.22%.

### 2.2. Lipase SMG1-Catalyzed Hydrolysis of Lower Glycerides in TAG Products

In order to obtain highly pure CLA-enriched TAG products, the above reaction mixture were further purified, and the lipase SMG1 was employed to hydrolyze lower glycerides (MAGs and DAGs) in the reaction mixture. The lipase SMG1-catalyzed hydrolysis process was optimized by response surface methodology.

#### 2.2.1. Model Analysis to Estimate the Coefficients

The CCRD was adopted in this study to identify the importance of the variables (water content, pH and reaction temperature) in the hydrolysis reaction. The experimental design matrix and the corresponding results are shown in Table 1. By applying regression analysis on the experimental data, the quadratic regression equations model is as follows (Equation 1), representing the TAG purity expressed as a function of variables:

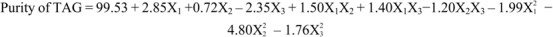
(1)
where X_1_, X_2_, and X_3_ are the water content, pH and reaction temperature, respectively.

**Table 1:** Experimental design and results of CCRD optimization experiment ^a^.

Trials	*X*_1_	*X*_2_	*X*_3_	Purity of TAG (%)	
				Observed	Predicted
1	−1(30)	−1(5)	−1(20)	91.5 ± 0.14	91.47
2	1(90)	−1(5)	−1(20)	91.3 ± 0.17	91.37
3	−1(30)	1(6)	−1(20)	92.3 ± 0.11	92.31
4	1(90)	1(6)	−1(20)	98.3 ± 0.13	98.21
5	−1(30)	−1(5)	1(30)	86.3 ± 0.10	86.37
6	1(90)	−1(5)	1(30)	91.9 ± 0.16	91.87
7	−1(30)	1(6)	1(30)	82.5 ± 0.17	82.41
8	1(90)	1(6)	1(30)	93.9 ± 0.12	93.92
9	−1.68(9.5)	0(5.5)	0(25)	89.1 ± 0.09	89.12
10	1.68(110.4)	0(5.5)	0(25)	98.7 ± 0.11	98.71
11	0(60)	−1.68(4.6)	0(25)	84.8 ± 0.14	84.75
12	0(60)	1.68(6.3)	0(25)	87.1 ± 0.14	87.18
13	0(60)	0(5.5)	−1.68(16.6)	98.5 ± 0.11	98.51
14	0(60)	0(5.5)	1.68(33.4)	90.6 ± 0.13	90.61
15	0(60)	0(5.5)	0(25)	99.5 ± 0.08	99.53
16	0(60)	0(5.5)	0(25)	99.5 ± 0.11	99.53
17	0(60)	0(5.5)	0(25)	99.7 ± 0.12	99.53
18	0(60)	0(5.5)	0(25)	99.4 ± 0.10	99.53
19	0(60)	0(5.5)	0(25)	99.5 ± 0.13	99.53
20	0(60)	0(5.5)	0(25)	99.6 ± 0.12	99.53

^a^ *X*_1_, water content (w/w, with respect to total oil mass); *X*_2_, pH value; *X*_3_, reaction temperature (°C).

Statistical experimental design techniques have been proved to be very useful tools for process optimization as they can not only complete optimization but also provide statistical models that assist in understanding the interaction of different variables [14]. The ANOVA was performed for the response surface quadratic model and the results are shown in Table 2. From the P-values of terms in Table 2, it can be seen that all of the terms, including the linear and square terms of water content, pH, temperature, and the interaction of the water content and pH, interaction of water content and reaction temperature and interaction of reaction temperature and pH, were highly significant on the TAG purity (*p* <0.05). The mathematical model was very reliable, with an R2 value of 0.9999. The closer R2 is to 1, the better the model fits the experimental data, and the less the difference between the predicted and the observed values. The low CV value of 0.10% indicated the great degree of precision with which the treatments were compared. The computed F value of 7728.11 was much greater than the *F*_(9, 10)_ value in statistical tables at a 1% level. It reflected the significance of the model.

#### 2.2.2. Main Effects and Interaction between Parameters

**Figure 2:**
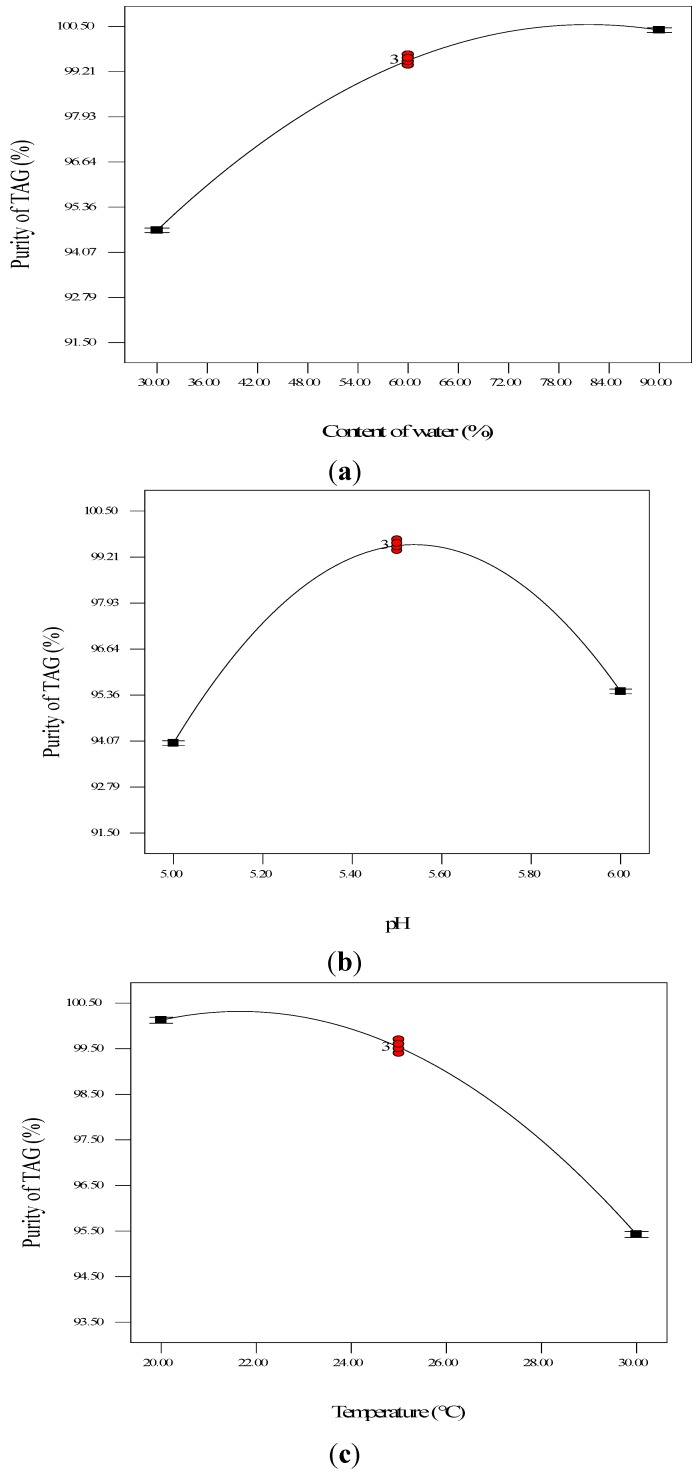
Main effect plot showing the effect of water content (**a**), pH (**b**) and temperature (°C) on TAG purity.

Since all three factors including water content, pH and reaction temperature affect the hydrolysis of lower glycerides significantly, the effect of these parameters on TAG purity was elucidated in detail and the results are shown in Figure 2. Effect of water content on the TAG purity increased dramatically with the water content increasing from 30% to 60% and thereafter, further increase in water content did not significantly improve the TAG purity (Figure 2a). It might result from the dilution effect on biocatalyst caused by excessive water [15]. The pH plays an important role in the lipase catalyzed hydrolysis of lower glycerides and thus significantly affected the TAG purity. As shown in Figure 2b, the TAG purity in the reaction products increased dramatically when the pH increased from 5 to 5.5 while an obvious decrease of TAG purity was found when pH was further increased to 6.0. Reaction temperature also has a significant effect on the lipase activity as well as the lipase catalyzed hydrolysis. As presented in Figure 2c, TAG purity decreased slightly with the temperature increasing from 20 to 25 °C while when the temperature further increased, the TAG purity decreased dramatically due to the denaturation of protein by the high temperature. In our previous study, the optimum temperature for maximum lipase activity of lipase SMG1 is between 25 and 30 °C [16], and the highest TAG purity was obtained at 20 °C which was consistent with the former results.

**Table 2:** Model-fitting results and analysis of variance for the TAG purity ^a^.

Source	Degree of freedom	Mean square	*F* value	Prob > *F*
Model	9	69.21	7728.11	<0.0001
X_1_	1	111.06	12400.74	<0.0001
X_2_	1	7.13	796.18	<0.0001
X_3_	1	75.38	8417.34	<0.0001
X_1_X_2_	1	18.00	2009.85	<0.0001
X_1_X_3_	1	15.68	1750.80	<0.0001
X_2_X_3_	1	11.52	1286.30	<0.0001
X_1_^2^	1	56.89	6352.52	<0.0001
X_2_^2^	1	331.71	37038.25	<0.0001
X_3_^2^	1	44.49	4968.01	<0.0001
Residual	10	8.956E-003		
Lack of Fit	5	7.245E-003	0.68	0.6592
Pure Error	5	0.011		

^a^ R^2^ = 0.9999, Adj R^2^ = 0.9997, *F*_(9, 10)_ = 4.94.

**Figure 3:**
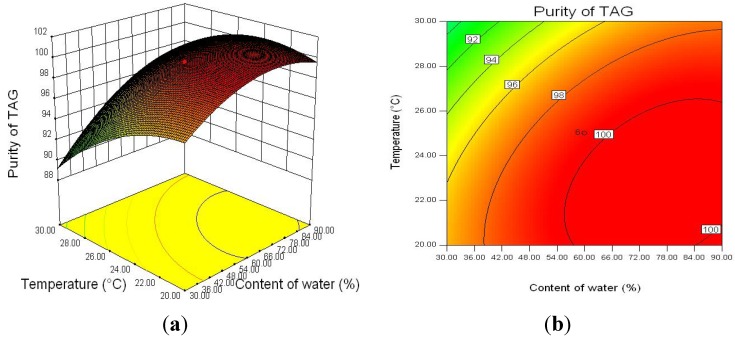
Response surface plot (**a**) and its contour plot (**b**) of the TAG purity; temperature *vs.* water content with a constant level of pH (pH = 5).

**Figure 4:**
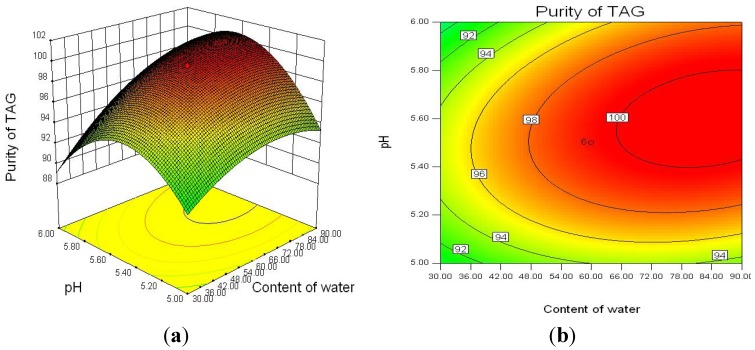
Response surface plot (**a**) and its contour plot (**b**) of the TAG purity; pH *vs.* water content with a constant level of temperature (20 °C).

**Figure 5:**
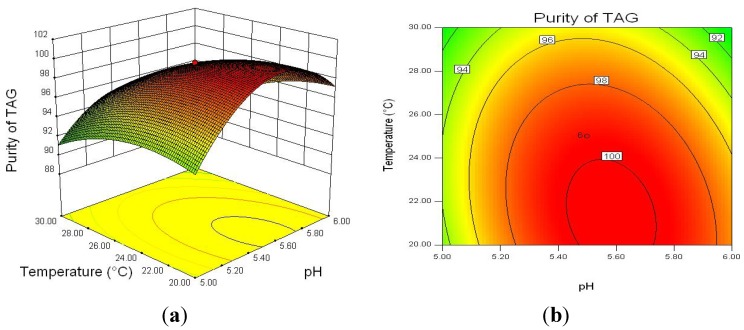
Response surface plot (**a**) and its contour plot (**b**) of the TAG purity; temperature *vs.* pH with a constant level of water content (60%).

To investigate the interaction between three factors on TAG purity, the response surface curves and the contour curves for the water content, pH, and reaction temperature are shown in Figure 3, Figure 4 and Figure 5. The response surface representing TAG purity was a function of two factors with another variable fixed at an constant level. The optimal levels and interaction between two factors were clearly revealed. Based on the response surface curve which was constructed for center level of temperature and water content (Figure 3), it is observed that the TAG purity increased with increasing the water content and decreasing the reaction temperature and higher water content and lower reaction temperature can improve the TAG purity. The interaction between pH and water content as well as that between pH and reaction temperature also had significantly effects on TAG purity (Figure 4 and Figure 5). From the response surfaces, it indicated that the optimal pH value of the buffer solution was about 5.5 for lower glycerides hydrolysis and the lower or higher pH decreased the TAG purity.

#### 2.2.3. Optimization of Enzymatic Hydrolysis Parameters for TAG Purity

According to the mathematical model, the optimal levels of the three factors were: water content 66.4%, pH 5.7 and reaction temperature 19.6 °C, the maximal TAG purity was 100%. The optimal values obtained from response surface plots were consistent with those obtained from the optimized mathematical equation. The experiment was performed under above optimized conditions to verify the predicted results. After 1 h of hydrolysis, there were only 0.3% of lower glycerides remained in the reaction mixture and the actual observed TAG purity reached to 99.7%. The results indicated that experimental values of TAG purity had a good agreement with the predicted value.

### 2.3. Purification of TAG by Molecular Distillation

The reaction mixtures were purified using a short path falling film distillation apparatus. The acylglycerol profile and fatty acid composition of purified TAG are shown in Table 3. When the reaction mixture was purified by molecular distillation, the FA was effectively removed, and the most of TAG was successfully collected from the residual fractions because of relatively larger molecular weight of TAG. The final TAG product, which is CLA-enriched TAG, was composed of 99.8% TAG and 0.2% DAG. The total yield of CLA-enriched TAG was 38.1%. In terms of fatty acid composition, the product did not result in significant changes in fatty acid composition of origin and final total CLA, and the content of t10, c12-CLA small increased and c9, t11-CLA decreased than origin. Previous study has reported that the NOVO 435 lipase selective esterified t10, c12-CLA faster than c9, t11-CLA [17]. The CLA content in the final TAG products was 80.20%. The PV of final product was 0.34 ± 0.04 meq/kg, and the FFA was not found.

**Table 3:** Acylglycerol profile, fatty acid composition of extremely pure CLA-enriched TAG.

	CLA	CLA-enriched TAG
Acylglycerol Profile (%)		
TAG	0	99.8
DAG	0	0.2
MAG	0	0
FA	100	0
Fatty Acid Compositions (%)		
C16:0	4.74	4.95
C18:0	2.22	2.32
C18:1	11.47	11.56
C18:2	0.95	0.97
Total CLA	80.62	80.22
c9,t11-CLA	33.95	31.78
t10,c12-CLA	43.59	44.92
Other CLA	3.08	3.52

## 3. Experimental

### 3.1. Materials

Novozym 435 (*Candida antarctica* B lipase immobilized on a macroporous resin) was purchased from Novozymes (Bagsvaerd, Denmark). The lipase SMG1 (60 U/mL free liquid lipase, lipase activity was determined according to Wang *et al.* [16]) was produced in lab as our previous report [16]. CLA was purchased from Aohai Biotech Company (Qingdao, China). CLA fatty acid compositions were 4.74% C16:0, 2.22% C18:0, 11.4% C18:1, 0.95% C18:2, 80.62% CLA. Chromatographic grade isopropanol, *n*-hexane, the standards of 1(3)-monooleoyl-rac-glycerol (CAS no. 111-03-5), 1,3-dioleoylglycerol (CAS no. 2465-32-9), 1,2-dioleoylglycerol (CAS no. 2442-61-7) and trioleoylglycerol (CAS no. 122-32-7) for HPLC analysis reference and 37 FA methyl esters (CAS no.113 47885-U) for GC analysis reference were acquired from Sigma Aldrich (St. Louis, MO, USA).

### 3.2. Preparation of CLA Glycerides

CLA-enriched triacylglycerol (TAG) were produced by direct esterification of glycerol and CLA using an immobilized lipase from *Candida antarctica* under vacuum according to the condition reported by Hong *et al.* [18] Reactions were performed in a 2 L water-jacketed glass vessel containing glycerol (100 g, 1.08 mol) and CLA (900 g, 3.21 mol). The immobilized lipase (10 g, 1% of the total weight of substrates) was then added to the mixture. The reaction was initiated with stirring at 600 rpm, 0.1 kPa and 60 °C. After 6 h of esterification, the reaction mixture was separated by centrifugation at 6,000 × *g* for 10 min. The upper mixture of glycerides and fatty acids were obtained.

### 3.3. Experimental Design

The response surface methodology (RSM) was performed to optimize the condition of variables for the hydrolysis of lower glycerides using a central composite rotatable design (CCRD). The water content (*X*_1_), pH value (*X*_2_), and reaction temperature (*X*_3_) were identified as the major factors. A set of 20 experiments were performed with the first 14 trials organized in a functional factorial design and with the later 15–20 trials relating to replication of the central points. The purity of TAG was taken as the response and the design matrix is shown in Table 1. The quadratic equation (Equation 2) for predicting the variables is as follows:

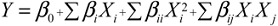
(2)
where *Y* is predicted response, *β*_0_ is a constant, *β*_i_, *β*_ii_ and *β*_ij_ are linear, squared and cross-product coefficient, respectively.

The statistical software Design Expert 8.0 was used to analyze the results. By keeping one variable at its optimal level, three-dimensional plots of two factors versus the TAG purity were drawn. Form the bump of three-dimensional plot, and the optimal conditions of enzymatic hydrolysis of lower glycerides were identified.

### 3.4. Lipase SMG1-Catalyzed Hydrolysis of Lower Glycerides

The hydrolysis reaction was carried out in a 25 mL conical flask with stirring at 180 rpm. The reaction mixture was composed of esterification product, lipase solution and different amount of distilled water. The reaction conditions of water content (30% and 90%, w/w, with respect to oil), pH value (5.0 and 6.0) and reaction temperature (20 and 30 °C) were varied to investigate their effects on the degree of hydrolysis of lower glycerides (referring to monoacylglycerol and diacylglycerol). CLA could be oxidized for longer reaction time, and the partial glyceride would not be hydrolyzed completely for shorter reaction time. Therefore, reaction time was fixed to 1h. Aliquots (20 µL) of the reaction mixture were periodically withdrawn from the reactions and then were mixed with 1 mL of *n*-hexane/isopropanol (15:1, v/v), after centrifugation at 10,000 × *g* for 2 min, the supernatant liquid was drawn for HPLC analysis.

### 3.5. Purification of the TAG by Molecular Distillation

The non-polar layer (TAG, FA, and/or MAG, DAG) of the final reaction mixture from the hydrolysis process was collected after centrifugation at 2,770 × *g* for 10 min. In order to obtain pure CLA-enrich TAG, the FFA in the mixture was removed by a molecular distillation (MD-S80 short path falling film distiller, Guangzhou Hanwei Co., Ltd., Guangzhou, China). An evaporating temperature of 150 °C, a feeding temperature of 60 °C, respectively, a feed flow rate of 2.7 g/min, a condenser temperature of 25 °C, a pressure of 8.1 Pa and a scraper speed of 250 rpm were used for the removal process. The residues with TAG and/or DAG, MAG were collected for HPLC analysis.

### 3.6. HPLC Analysis of the Products

The content of TAG and FFA in the ersterification products, hydrolytic products and the residue oil after the molecular distillation was analyzed by a Waters 2695 HPLC with a parallax refractive index detector on a Phenomenex Luna silica column (Phenomenex Corporation, Torrance, CA, USA, 250 mm × 4.6 mm i.d., 5 µm particle size). The mobile phase was a mixture of *n*-hexane and isopropanol (15:1 v/v) with a flow rate of 1.0 mL/min. Peaks in HPLC were identified by comparison of their retention times with reference standards. Peak-areas percentages were calculated using Waters 2695 integration software. The product mixture of esterification reaction contains glycerol, FFA, MAG, DAG and TAG. Glycerol and FFA can be removed easily by molecular distillation. And the DAG and MAG cause the difficulty for purifying TAG, because of the small vapor pressure difference between DAG and TAG. It is necessary to reduce the content of DAG and MAG. Thus, TAG purity was defined as Equation 3.


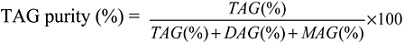
(3)

### 3.7. Analysis of Fatty Acid Compositions of TAGs

The TAG fraction in the esterification products was separated by a thin-layer chromatography (TLC) plate (100 × 200 mm) coated with silica gel and was developed in a TLC tank using petroleum ether/ethyl ether/acetic acid 80/20/1 (v/v/v). The bands were visualized with 0.2% 2,7-dichlorofluorescein in methanol under ultraviolet light and the TAG band was scraped off for the analysis of FA composition.

The TAGs were transmethylated according to ISO 5509:2000(E) [19]. Samples were added to a round-bottom flask for methyl transesterification and then were separated on a FFAP column (PERMABOND-FFFAP DF-0.25, 25 m × 0.25mm i.d., Macherey-Nagel, Düren, Germany) by an Agilent 7890 GC (Agilent Tech, Santa Clara, CA, USA) using nitrogen as the carrier gas. A temperature program was used to keep the samples in a column oven at 150 °C for 2 min. The temperature was increased to 230 °C at 10 °C/min and held for 8 min for a total run time of 18 min. The split ratio was 50:1. The injector and the flame ionization detector temperatures were set at 250 and 300 °C, respectively.

### 3.8. Other Analyses

The free fatty acid (FFA) of the test oils was determined with the alkali titration method [20]. The peroxide value (PV) was determined spectrophotometrically by the International Dairy Federation (IDF) method [21].

### 3.9. Statistics

All analytical determinations were carried out in triplicate. The results are reported as the means ± standard deviations (SD) of these measurements.

## 4. Conclusions

In summary, the designed synthesis method providea useful process for the production of extremely pure CLA-enriched TAG. Due to the thermal instability of CLA, high temperature molecular distillation (220–250 °C) cannot be applied to separate lower glycerides from TAG, which will still exist in the final products and result in the low TAG purity. The presented method provides an alternative process to separate the lower glycerides by lipase SMG1-catalyzed hydrolysis.

RSM was successfully adopted to model and optimize the hydrolysis of lower glycerides and main effects and interactions between the three reaction parameters (water content, pH and reaction temperature) were fully elucidated. The optimized conditions by lipase SMG1-catalyzed hydrolysis of lower glycerides were as following: water content of 66.4% (w/w, with respect to oil mass), pH at 5.7 and 1 h of reaction time at 19.6 °C. Based on the optimal conditions, only 0.3% of lower glycerides remained in the reaction mixture and the purity of CLA-enriched TAG reached to 99.7%. A lower temperature molecular distillation (150 °C) was employed to separate the FFA from TAG and the final CLA-enriched TAG product yielded 99.8% of TAG. By applying this method, extremely pure CLA-enriched TAG can be easily obtained at a purity of over 98% for use in studies of the CLA metabolism in animals and humans.
